# An unusual case of acute pseudogout in the hand masquerading as cellulitis

**DOI:** 10.1097/MD.0000000000046396

**Published:** 2026-01-02

**Authors:** Yake Meng, Lili Zhou, Rongxue Shao, Wei Zhag, Hao Pan

**Affiliations:** a Department of Orthopedics, Hangzhou Hospital of Traditional Chinese Medicine, Zhejiang Chinese Medical University, Hangzhou, Zhejiang Province, China; b Department of Neurology, Hangzhou Hospital of Traditional Chinese Medicine, Zhejiang Chinese Medical University, Hangzhou, Zhejiang Province, China.

**Keywords:** calcium pyrophosphate deposition, cellulitis, hand, pseudogout

## Abstract

**Rationale:**

The wrist joint represents one of the most commonly affected anatomical sites in calcium pyrophosphate deposition disease, yet it remains underreported in the literature, frequently leading to diagnostic errors.

**Patient concerns:**

We present a case of acute pseudogout of the wrist whose symptoms mimicked cellulitis. Our patient presented with a swollen hand, which was initially treated as acute cellulitis with antibiotics, but without any improvement.

**Diagnoses:**

A final diagnosis of acute chondrocalcinosis was made based on multiple intra-articular calcifications.

**Outcomes:**

The patient’s symptoms were significantly relieved, and her inflammation markers were markedly reduced after glucocorticoid administration.

**Lessons:**

Pseudogout of the wrist can present with a pseudocellulitis appearance in the acute phase. Early accurate diagnosis can help physicians provide adequate treatment and avoid unnecessary antibiotic use.

## 1. Introduction

Calcium pyrophosphate dihydrate deposition disease is one of the most common types of inflammatory arthritis, primarily affecting the elderly. The prevalence of calcium pyrophosphate deposition disease (CPPD) increases with age, rising from approximately 15% in individuals over 60 to nearly 30% in those over 80.^[1]^ The knee was the most commonly involved joint in CPPD, followed by the wrist. The small joints of the hands can also be affected; however, the relevant literature on this presentation is scarce. The clinical manifestations of CPPD disease span a broad spectrum, from asymptomatic presentation to severe, destructive polyarticular arthritis.^[2]^ This nonspecific clinical picture frequently complicates accurate diagnosis. We herein report a case of acute pseudogout affecting the hand in an elderly woman, which was initially misdiagnosed as cellulitis.

## 2. Case report

In March 2024, a 71-year-old woman presented with an erythematous, warm, and swollen left hand for the past 3 days. The patient denied a recent history of hand trauma. She suffered from a history of unexplained left forearm and hand swelling 2 years ago. Prior diagnosis and treatment details were unknown. She had a significant medical history that included diabetes, high blood pressure, and osteoarthritis of the knees. She was currently taking metformin and amlodipine. There was no family history of gout.

**Figure 1.**
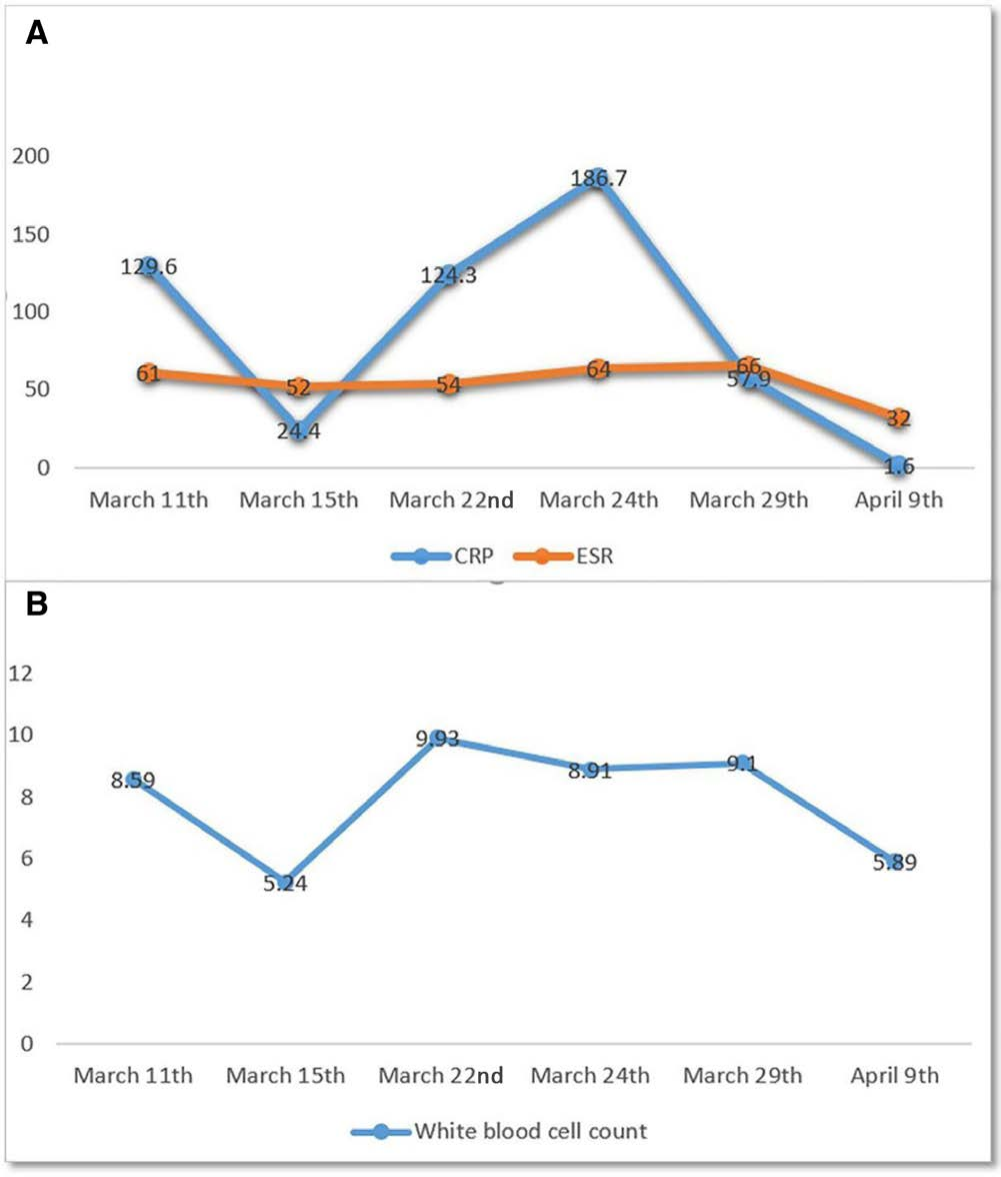
(A) Dynamic change process of inflammatory markers. (B) Dynamic change of white blood cell counts (WBC) during the treatment. CRP = C-reactive protein, ESR = erythrocyte sedimentation rate, WBC = white blood cell count.

**Figure 2.**
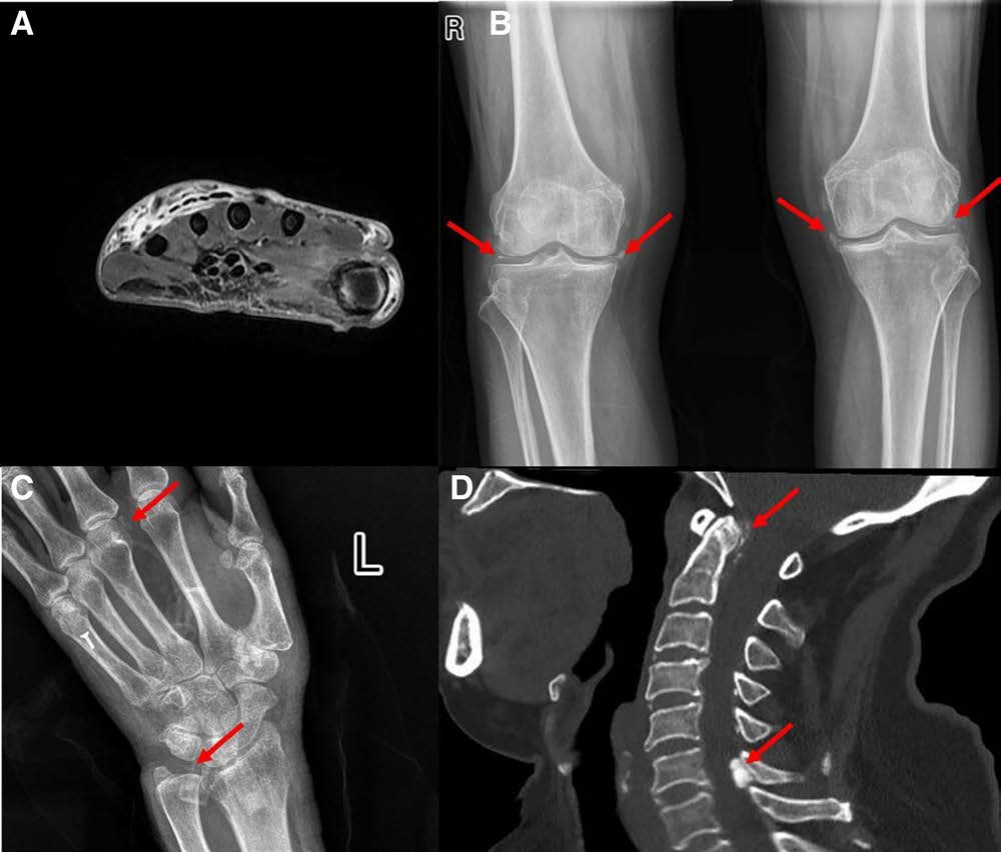
(A) MRI of the left hand showing diffuse soft tissue swelling. (B) X-ray of the knees showed bilateral osteoarthritis with linear calcification of the menisci in both knees. (C) Left wrist X-ray showed calcification of the triangular cartilage and calcification around the third metacarpophalangeal joint. (D) CT images of the cervical spine showed calcification of both the transverse ligament and the ligamentum flavum. CT = computed tomography, MRI = magnetic resonance imaging.

The clinical examination revealed severe diffuse swelling, redness, and tenderness of the left hand, and the range of motion of the hand joints was markedly decreased. Her peripheral pulse was palpable, and no sensory or motor deficits were detected. She had a 36.2°C temperature. She was admitted to the hospital (March 11, 2024), and her initial workup revealed a significantly elevated C-reactive protein 129.6 mg/L (<10 mg/L) with an erythrocyte sedimentation rate of 61, and a normal white cell count of 8.59 × 10^6^/L (<9.5 × 10^6^/L) (Fig. 1). No obvious abnormality was observed in the blood biochemical test. A diagnosis of hand cellulitis was made, and she was started on intravenous antibiotics, pain medication, intravenous antibiotics amoxicillin, glycerol fructose, and oral administration of loxoprofen sodium tablets. Three days after initiation of treatment, there was substantial improvement in the patient’s clinical status. Consistent with this, magnetic resonance imaging shows a bone bruise, soft-tissue edema, and C-reactive protein came down to 24.4 mg/L (March 15th, Fig. 1). Anti-infective therapy was continued, wrist joint function was significantly improved, whereas a rebound inflammation was noted (March 22nd, Fig. 1). What’s worse, approximately 13 days after initiation of treatment, the patient’s condition deteriorated with fever, and ankle joint pain, elevating inflammatory markers (March 24th, Fig. 1). Rheumatology and infectious disease teams were consulted. Antinuclear antibody and rheumatoid factor tests were done, which were all negative. Amoxicillin was discontinued and replaced by amoxicillin clavulanate potassium. Ultrasound of the wrist and ankle joints revealed synovial hyperplasia. Unfortunately, both treatments failed to improve symptoms and lower the inflammatory markers in the patients. A detailed medical history was taken again, and the patients’ medical records from previous hospital visits were collected, which included cervical spine magnetic resonance imaging, wrist and hand X-ray, and dual-energy computed tomography (CT) of the wrist. But the radiological pictures, therapeutic process, and final diagnosis were not obtained. Based on our own experience and the patient’s medical records of polyarthralgia, the presented patient underwent multiple imaging examinations, including X-ray of the knees and left wrist, and CT images of cervical spine. X-ray of the knees showed bilateral osteoarthritis with linear calcification of the menisci in both knees. Left wrist X-ray showed calcification of the triangular cartilage and calcification around the third metacarpophalangeal (MCP) joint. CT images of the cervical spine showed calcification of both the transverse ligament and the ligamentum flavum (Fig. 2).

**Figure 3.**
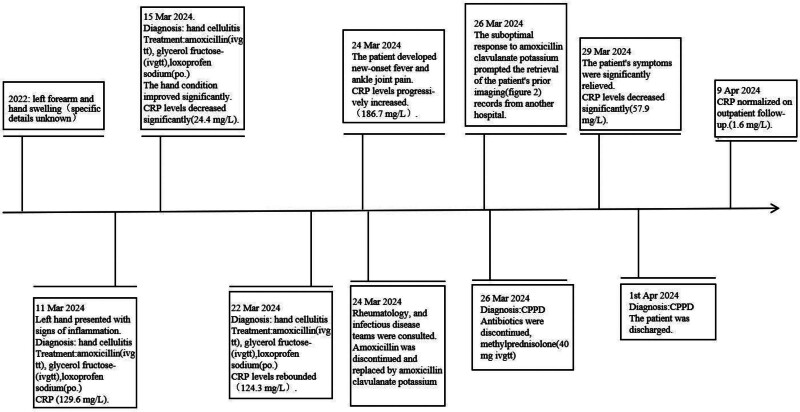
Diagnosis and treatment process of the patient. CPPD = calcium pyrophosphate deposition disease, CRP = C-reactive protein.

A diagnosis of acute CPPD, formerly pseudogout, was considered. Antibiotics were discontinued, and systemic glucocorticoids were administered. Methylprednisolone (40 mg) was infused intravenously for 3 consecutive days, and the methylprednisolone dose was halved on the 4th day. The patient’s symptoms were significantly relieved and her inflammation markers were markedly reduced (March 29th). The patient was later discharged on tablet methora (8 mg/day) for 3 days, and then was tapered off. One week after discharge, her clinical symptoms and inflammatory markers resolved to the normal range (April 9th) (Fig. 3).

## 3. Discussion

Acute calcium pyrophosphate crystal (CPP) arthritis (pseudogout or acute chondrocalcinosis) encompasses a form of acute crystal-induced arthritis, characterized by fever and marked systemic inflammation.^[3]^ This condition is typically monoarticular and predominantly affects elderly women. The knee is the most frequently involved joint, followed by the wrist. Although pseudogout can involve the MCP joints, such cases are scarcely documented in the English literature. The disease frequently poses a diagnostic challenge in its early phases by mimicking inflammatory or infectious conditions, which can result in misdiagnosis or failure to recognize the true etiology.

Pseudogout masquerading as septic arthritis has been documented in several case reports.^[4,5]^ However, only isolated cases of pseudogout clinically mimicking cellulitis have been reported. Awan et al^[6]^ reported a case of acute pseudogout in the wrist joint that masqueraded as cellulitis. The patient presented with acute swelling of the left forearm and hand that was refractory to antibiotic therapy. The definitive diagnosis of acute chondrocalcinosis was established by the identification of positively birefringent, rhomboid-shaped crystals in the synovial fluid (SF) aspirated from the wrist joint. Rana et al^[7]^ reported 2 cases of acute calcium pyrophosphate dihydrate arthritis that were initially misdiagnosed as cellulitis. In our patient, given the clinical presentation, a prior history of left forearm and hand swelling (despite incomplete details), and relevant laboratory findings, an initial diagnosis of hand cellulitis was made. The patient’s clinical and laboratory findings promptly improved upon starting anti-infective therapy. Despite ongoing anti-infective therapy, a paradoxical rise in inflammatory markers and recurrence of symptoms occurred in the later treatment stage. Radiographic demonstration of calcifications across multiple joint sites guided the final diagnosis of CPPD. The patient subsequently experienced prompt resolution of symptoms and normalization of inflammatory markers with steroid treatment.

Pseudogout is a clinical manifestation of calcium pyrophosphate deposition, typified by recurrent, self-limiting attacks of acute arthritis with associated joint swelling and pain. The diagnostic gold standard for pseudogout – identification of CPP in SF – has limited utility when joint aspiration is impractical, especially in small joints. When SF analysis for CPP crystals is unavailable, the typical radiographic appearance of chondrocalcinosis at characteristic sites can provide supportive diagnostic value.^[8]^ Radiographic examination of our patient revealed linear calcifications at multiple joint sites, including the bilateral knees, left wrist, left third MCP joint, and cervical spine (involving the transverse ligament and ligamentum flavum), and the diagnosis of CPPD was established.

Skeete et al^[9]^ found that gout, pseudogout, and cellulitis were the most common causes of suspected wrist joint infections. Clinical suspicion is important for early diagnosis. Based on our clinical experience, we propose that CPPD be considered in cases of acute nontraumatic erythema and swelling of the hands or forearms that do not respond to antibiotics, particularly in elderly women without gout. A critical consideration is that the existing therapeutic arsenal for CPPD remains confined to symptom management. First-line management of acute CPPD attacks includes colchicine, nonsteroidal anti-inflammatory drugs, or corticosteroids. In contrast, asymptomatic CPPD typically does not require any therapeutic intervention.

## 4. Conclusions

As an increasingly common form of arthritis in the elderly, CPPD also warrants note as one of the common differential diagnoses for suspected wrist joint infections. We herein describe what we believe to be the first case of recurrent wrist CPPD that was initially misdiagnosed as cellulitis, highlighting a potential diagnostic pitfall. This report highlights that CPPD can mimic cellulitis in its acute phase, making accurate and early diagnosis essential for directing appropriate management and avoiding superfluous antibiotic treatment.

## Author contributions

**Formal analysis:** Yake Meng.

**Investigation:** Yake Meng.

**Conceptualization:** Lili Zhou.

**Data curation:** Lili Zhou.

**Validation:** Rongxue Shao.

**Writing—original draft:** Rongxue Shao, Wei Zhag.

**Visualization:** Wei Zhag.

**Writing—review & editing:** Wei Zhag.

**Software:** Hao Pan.

**Supervision:** Hao Pan.
